# Análisis bibliométrico de la producción científica latinoamericana sobre COVID-19

**DOI:** 10.7705/biomedica.5571

**Published:** 2020-11-13

**Authors:** Orlando Gregorio-Chaviano, César H. Limaymanta, Evony K. López-Mesa

**Affiliations:** 1 Departamento de Ciencia de la Información, Facultad de Comunicación y Lenguaje, Pontificia Universidad Javeriana, Bogotá, D.C., Colombia Pontificia Universidad Javeriana Departamento de Ciencia de la Información Facultad de Comunicación y Lenguaje Pontificia Universidad Javeriana BogotáD.C Colombia; 2 Línea de Estadística, Departamento de Ciencias, Universidad Peruana de Ciencias Aplicadas, Lima, Perú Universidad Peruana de Ciencias Aplicadas Departamento de Ciencias Universidad Peruana de Ciencias Aplicadas Lima Peru; 3 Coordinación de Servicios al Usuario, Biblioteca, Universidad La Gran Colombia, Bogotá, D.C., Colombia Universidad La Gran Colombia Universidad La Gran Colombia BogotáD.C Colombia

**Keywords:** infecciones por coronavirus, bibliometría, indicadores bibliométricos, gestión de la información, América Latina, Coronavirus infections, bibliometrics, bibliometric indicators, information management, Latin America

## Abstract

**Introducción.:**

La propagación de la COVID-19, una enfermedad infecciosa causada por el nuevo coronavirus SARS-CoV-2, se ha convertido en una pandemia que, a la par de su rápida diseminación a nivel mundial, ha traído consigo un aumento exponencial de la cantidad de estudios relacionados con el tema, fenómeno en el que los investigadores de Latinoamérica han participado activamente.

**Objetivo.:**

Llevar a cabo un estudio bibliométrico descriptivo para identificar las tendencias de la investigación sobre COVID-19 producida en Latinoamérica.

**Materiales y métodos.:**

Se recurrió a las bases de datos Web of Science, Scopus y Pubmed para recuperar la producción científica latinoamericana sobre COVID-19. Se analizaron los indicadores bibliométricos de producción, visibilidad, impacto y colaboración para evaluar la participación regional en la investigación sobre el tema.

**Resultados.:**

El análisis de 142 documentos evidenció un crecimiento exponencial de la producción científica en el corto periodo analizado, una significativa colaboración internacional (51,4 %), y el liderazgo de las instituciones regionales (71 %) en la investigación con aportes en revistas de alta visibilidad, especialmente de Colombia, Brasil y México.

**Conclusiones.:**

El estudio evidenció resultados relevantes sobre la participación regional en la investigación sobre COVID-19, no solo en cuanto a la cantidad y el crecimiento exponencial, sino también a su calidad y excelencia, con una elevada tasa de colaboración internacional y de publicación en revistas de reconocido prestigio, lo que, además de ser clave para la visibilidad de los países, es un considerable aporte a las investigaciones que se realizan en otros contextos geográficos.

## Abstract (en)

**Introduction:**

The propagation of COVID-19, an infectious disease caused by the novel SARS-CoV-2 coronavirus, has become a pandemic which, along with its rapid dissemination worldwide, has brought about an exponential increase in the amount of research related to the subject to which Latin American researchers have contributed actively.

**Objective:**

To conduct a descriptive bibliometric study of the main trends in research on COVID-19 produced in Latin America.

**Materials and methods:**

We searched in the Web of Science, Scopus, and Pubmed databases to retrieve the Latin American scientific production on COVID-19. Bibliometric indicators of production, visibility, impact, and collaboration were analyzed to assess the regional participation in studies on the subject.

**Results:**

The analysis of 142 documents evidenced an exponential growth of scientific production in the period analyzed, an important level of international collaboration (51.4%) in scientific production, and the leadership of regional institutions (71%) in the research with publications in high-visibility jounals especially in Colombia, Brazil, and México.

**Conclusions:**

The results regarding the regional participation in the research on COVID-19 were relevant not only in relation to its quantity and exponential growth during the period analyzed but also in terms of its quality and excellence with a high rate of international collaboration and publications in important scientific journals, which besides their visibility, represent a considerable contribution to the research compared to the other geographical contexts.

La situación global provocada por la COVID-19, enfermedad infecciosa causada por el nuevo coronavirus SARS-CoV-2, obligó a la Organización Mundial de la Salud (OMS) a declarar el estado de pandemia ^1^. La necesidad de encontrar posibles tratamientos para frenar la enfermedad ha llevado al aumento de la producción científica sobre el tema. En este contexto, la colaboración entre países e instituciones y el trabajo coordinado que aporte soluciones para la prevención y mitigación de la pandemia cobran especial relevancia ^2^. En este sentido, se han emitido recomendaciones encaminadas al fortalecimiento de las políticas de emergencia de salud pública y se ha reforzado el trabajo en cada país para determinar los factores de riesgo y hacerle seguimiento a su comportamiento, dando cumplimiento a las normas sanitarias internacionales.

El crecimiento y la expansión de esta enfermedad desde diciembre de 2019 han traído consigo un aumento considerable de la producción científica a nivel mundial en diferentes frentes de trabajo debido a la necesidad de encontrar soluciones sanitarias y controlar la enfermedad y su avance en los países afectados ^3^.

A partir de la necesidad de soluciones que frenen el avance de la enfermedad, un gran número de investigaciones e importantes editoriales como Elsevier, Taylor and Francis y Springer han dado acceso abierto a sus contenidos relacionados con la COVID-19 ^3^. Esta iniciativa busca incrementar el acceso a las nuevas investigaciones como soporte para la generación de conocimientos y la búsqueda rápida de soluciones.

Entre los innumerables estudios disponibles, los análisis bibliométricos han dado cuenta del ritmo acelerado de la producción científica, sus tendencias y regularidades, los muchos países e instituciones contribuyentes y los temas más tratados ^4^. También han descrito las principales tendencias de la producción existente, como las características de la enfermedad y los posibles tratamientos ^5^. Se ha evidenciado el liderazgo de China en la producción científica global, además de la representatividad de otros países como Estados Unidos, Francia y Alemania. Desde el punto de vista temático, es notable la orientación hacia la investigación de temas epidemiológicos y de virología ^6^.

En este marco, se hizo un estudio bibliométrico de corte descriptivo de la investigación latinoamericana sobre COVID-19, analizando la participación de la región a partir de indicadores bibliométricos. Al igual que en estudios recientes ^3^, se espera que los resultados que aquí se resumen contribuyan al desarrollo de investigaciones futuras y sirvan de guía en la gestión del control de la enfermedad. Aunque restringido a un periodo limitado, el análisis de la información registrada en una muestra de algunas de las más importantes fuentes de datos, ofrece resultados cuantitativos y revela regularidades y comportamientos de la temática, por lo que pueden ser punto de partida para análisis posteriores, además de un aporte a la gestión de las investigaciones sobre la enfermedad en las instituciones de Latinoamérica.

La pregunta de investigación se planteó en los siguientes términos: ¿cuáles son las principales tendencias en la producción científica latinoamericana sobre COVID-19?

## Materiales y métodos

Los documentos que sirvieron de fuente para el análisis bibliométrico provienen de las bases de datos Web of Science (SCI), Scopus y PubMed, la fecha de corte de la búsqueda fue el 23 de abril de 2020. Se empleó la ecuación de búsqueda para la extracción de documentos ("COVID-19" OR "2019-nCoV" OR "SARS-CoV-2" OR "new coronavirus" OR "coronavirus disease 2019") en los campos de título, resumen y palabras clave, y las tipologías de 'Article"y "Review" en los países de la región. Se normalizaron las variables de autor, institución, país y palabras clave, dado que a partir de ellas se generaron los indicadores bibliométricos.

**Figura 1:**
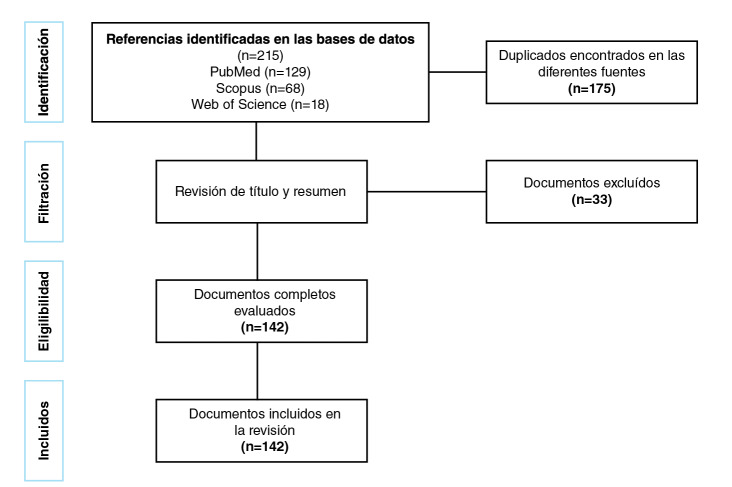
Proceso metodológico de búsqueda, recuperación y selección de la información para el análisis

Mediante esta estrategia de búsqueda se recuperaron 215 documentos, los cuales fueron sometidos a un proceso de normalización de metadatos y de eliminación de documentos duplicados. La muestra final para el análisis bibliométrico quedó compuesta por 142 documentos (figura 1).

Se empleó la herramienta bibliográfica EndNote 7.0 para el procesamiento de los datos, en tanto que las listas de distribución de frecuencia se generaron con los programas Microsoft Excel 2016. Para el estudio del comportamiento de la productividad de los autores se recurrió al modelo inverso de Lotka (ley de Lotka), el cual permite conocer cuáles son los autores élite y transitorios en una disciplina ^7^^-^^9^, además, el modelo de Bradford para conocer las revistas más productivas que se encuentran en el núcleo ^10^ y el modelo de Price como instrumento para analizar el crecimiento acumulado de la producción científica ^11^.

Para estudiar la colaboración latinoamericana, se analizaron los patrones de colaboración y el liderazgo científico. Para complementar el estudio, se analizaron las redes de coautoría y de coocurrencia de palabras clave generadas con VOSviewer, vl.6.15, programa que permitió la visualización de mapas basados en la distancia de los nodos ^12^, además del método *fractional counting,* el cual se recomienda para este tipo de análisis dado que asigna el mismo peso a cada acción ^12^^,^^13^. Para una mejor comprensión de la metodología y los resultados, se exponen los principales indicadores analizados con su respectiva descripción (cuadro 1).

**Cuadro 1:** Descripción de los principales indicadores bibliométricos analizados de la producción científica sobre COVID-19

Indicador	Descripción
Comportamiento de la producción	Indica el comportamiento de la producción científica sobre COVID-19 en el periodo para conocer sus regularidades y tendencias. El modelo de Price permitió evaluar el ritmo de crecimiento de la producción científica.
Productividad de los autores	Evidencia si una menor cantidad de autores concentra el mayor volumen de la producción científica. Se emplea la ley de Lotka que permite conocer cuáles son los autores más importantes (élite), frente a los transitorios (poco especializados).
Producción por revistas	Establece las revistas fuente de la producción científica sobre COVID-19 y sus indicadores de visibilidad e impacto. Se utiliza el modelo de Bradford para conocer en un listado de productividad por revistas, aquellas de mayor número de documentos sobre el tema (núcleo), frente a las de mediana y escasa productividad (centro y periferia).
Patrones de colaboración	Los patrones de colaboración indican la forma en que los autores se relacionan en el proceso de escritura y da cuenta de la apertura de la investigación.
Liderazgo científico	Indica la participación latinoamericana en las investigaciones en cuanto a si se genera o no investigación en la región. Datos extraídos del autor de correspondencia de cada documento.
Red de coautoría	Se utiliza para determinar la forma en que los autores trabajan en red formando colegios invisibles.
Red de palabras clave	Indica cómo aparecen los principales descriptores en el conjunto de documentos analizados y permite analizar el enfoque temático y los frentes de investigación en la producción científica a partir de los clúster.

## Resultados

### Comportamiento semanal de la producción sobre COVID-19

**Figura 2:**
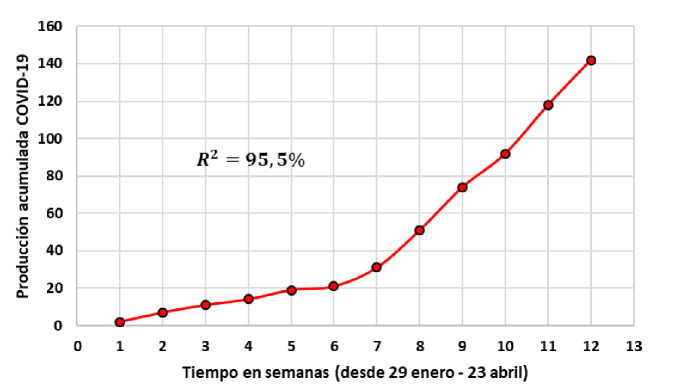
Modelo exponencial del crecimiento acumulado de la producción latinoamericana sobre la COVID-19

En la figura 2 se presenta la producción científica acumulada mediante el modelo exponencial de Price ^11^ con una tasa de crecimiento semanal de 41,3 % y con un índice de bondad de ajuste de R^2^=95,5 %. Este volumen de producción está registrado en 86 revistas científicas con la participación de 874 autores de diversas instituciones. La semana 1 inició el 29 de enero con un aumento sostenido en el crecimiento a partir de la séptima semana.

### Producción de los autores

**Figura 3:**
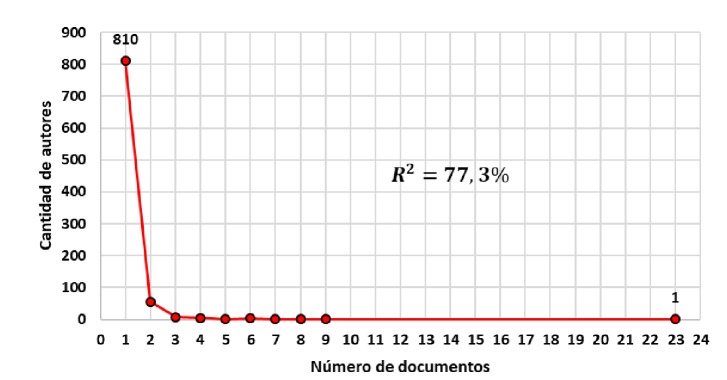
Productividad de autores según número de documentos publicados sobre COVID-19 con la ecuación del modelo inverso de Lotka

En la figura 3 se presenta la participación de los autores según el número de documentos publicados. Del total de 874 autores, 788 publicaron un solo documento, en tanto que un solo autor publicó 23 documentos. Se cumple, por lo tanto, que una cantidad reducida de autores concentra el mayor volumen de la producción científica, en tanto que la mayoría de ellos registra poca productividad.

Este fenómeno, conocido como el Efecto Mateo en la producción científica ^14^, refleja la existencia de un número pequeño de autores especializados que concentra el flujo de información y una gran cantidad de autores transitorios con pocas publicaciones. El modelo inverso de Lotka, cuyo coeficiente de determinación es de R^2^ =77,3 %, se ajusta a los datos analizados.

La importancia del modelo aplicado en el presente análisis es que permite conocer cuáles son esos investigadores élite que lideran la investigación sobre el tema.

En el cuadro 2 se evidencia la relación de los autores que hasta la fecha han aportado la mayor cantidad de estudios sobre COVID-19. Los 874 autores de los 142 documentos analizados se agrupan en pequeños, medianos y grandes productores según la ley de Lotka. Entre aquellos que han aportado más de tres documentos hasta la fecha, se destacan investigadores como Rodríguez-Morales, Bonilla-Aldana, Giovanetti y Balbin-Ramón, entre otros.

**Cuadro 2:** Autores latinoamericanos con la mayor producción de documentos sobre COVID-19

Autores	Número de documentos	Institución
Rodríguez-Morales AJ	23	Universidad Tecnológica de Pereira
Bonilla-Aldana DK	8	Fundación Universitaria Autónoma de las Américas
Giovanetti M	7	Fundação Oswaldo Cruz
Balbin-Ramón GJ	4	Hospital de Emergencias José Casimiro Ulloa, Perú
Franco-Paredes C	4	Hospital Infantil de México
García LP	4	Instituto de Pesquisa Económica Aplicada
Paniz-Mondolfi A	4	Instituto de Investigaciones Biomédicas, IDB
Zambrano LI	4	Universidad Nacional Autónoma de Honduras
Codeço CT	3	Fundação Oswaldo Cruz, Brasil
Croda JHR	3	Ministério da Saúde, Brasil

### Liderazgo científico y patrones de colaboración

El liderazgo científico de una institución se refleja en la cantidad de estudios cuyo investigador principal (autor de correspondencia) pertenece a ella y refleja la capacidad que tiene para generar proyectos de investigación ^15^ y, en este caso específico, de generar proyectos relacionados con la COVID-19. De los 142 documentos regionales, un alto porcentaje refleja el liderazgo latinoamericano (71 %) comparados con los de investigación participativa (como integrantes de proyectos de otras instituciones) (29 %), lo que evidencia la labor de las instituciones regionales y su capacidad para generar proyectos.

La coautoría como vía explícita de la colaboración permite el aumento de la visibilidad, del impacto de la investigación y la existencia de colegios invisibles, es decir, agrupaciones de investigadores y de grupos de investigación en torno a temas específicos. Existe una relación entre la calidad, el impacto, la relevancia científica y la cantidad de autores que aparecen en los textos, lo que es muy diferente entre las disciplinas científicas ^16^.

Un total de 874 autores participan en los 142 documentos obtenidos, lo que equivale a un índice de colaboración de la producción científica de 6,15 (promedio de autores firmantes por trabajo), y a un grado de colaboración de 0,87 (el 87 % de los documentos fue escrito por dos o más autores). Esta tendencia refleja la elevada participación de autores en las investigaciones, lo que responde no solo al comportamiento por disciplinas: las básicas y las aplicadas tienden a tener un mayor número de autores en los documentos ^17^, y a los diferentes hábitos de producción y citación ^18^, sino también a la necesidad de cooperación en las investigaciones sobre el tema analizado.

### Red de coautoría

**Figura 4:**
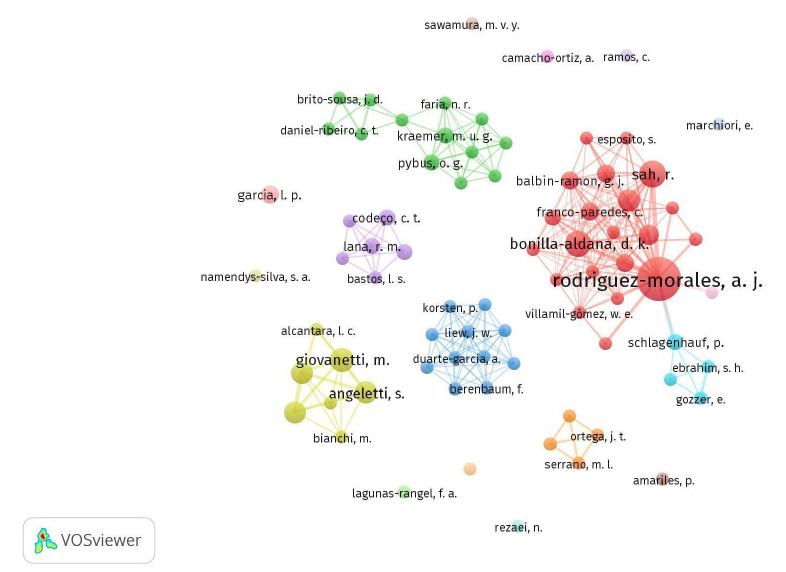
Visualización de una red de coautoría.

La coautoría, es decir, cuando dos o más autores deciden escribir un documento en colaboración, es la manifestación más clara de la colaboración científica y refleja los vínculos entre los investigadores. En la red de coautoría, los nodos o vértices representan a los autores y los vínculos son las relaciones en la producción de documentos ^19^, lo que refleja los autores que trabajan en red y los que trabajan en solitario. En la figura 4 se presenta el mapa de la red de coautoría obtenido con el programa VOSviewer, donde cada círculo (nodo) representa a un investigador.

Nota: se tuvieron en cuenta autores con dos documentos como mínimo (n=2). De los 887 autores, 77 cumplían con el umbral (8,7 %); método de conteo: fractional counting; método de normalización: fuerza de asociación; atracción: 2; repulsión: -3; resolución de agrupamiento: 0,5

El autor más productivo es A. J. Rodríguez-Morales, afiliado a la Universidad Tecnológica de Pereira y a la Universidad Autónoma de la Américas, ambas en Colombia, con 23 documentos publicados, seguido de D. K. Bonilla-Aldana, con ocho documentos. Según la red de coautoría, cuanto más cerca uno de otro se ubican los investigadores en la red, mayor es la relación de coautoría entre ellos, por ejemplo, Bonilla-Aldana con C. Franco-Paredes. Los colores indican agrupaciones de investigadores que están relativamente relacionados entre sí. De las 18 agrupaciones, 11 están conformadas solo por un autor, lo que evidencia la tendencia al trabajo individual por parte de algunos autores.

En el estudio se encontraron 120 documentos con colaboración, 75 de ellos (52,8 %) con colaboración internacional y 48 (33,8 %), con colaboración nacional. Se demostró un patrón determinante de colaboración internacional, comportamiento lógico, dada la urgencia del tema y la necesidad de compartir información y esfuerzos para el logro de vacunas y resultados positivos frente a la pandemia. Es de destacar, además, la presencia de 19 contribuciones de un solo autor (13,4 % del total de los 142 documentos).

### Productividad por institución

En cuanto a la productividad por institución, 546 instituciones internacionales han participado en la producción latinoamericana sobre COVID-19 y solo 21 (4 %) han producido más de cinco artículos. En el cuadro 3 se presentan los resultados de las instituciones con una frecuencia de publicación de cuatro o más documentos, entre los que se destacan las instituciones de Colombia, Brasil y México. Como dato de interés, el autor de mayor productividad (Rodríguez-Morales), ya mencionado, pertenece a las dos primeras instituciones.

**Cuadro 3:** Principales instituciones latinoamericanas que participan en la investigación sobre COVID-19

R	Institución	País	Número de documentos
1	Universidad Tecnológica de Pereira	Colombia	35
2	Fundación Universitaria Autónoma de las Américas	Colombia	29
3	Fundação Oswaldo Cruz	Brasil	18
4	University of São Paulo	Brasil	12
5	Instituto Nacional Ciencias Médicas Salvador Zubirán	México	10
6	Universidad Científica del Sur	Perú	8
7	Instituto Nacional de Salud Pública, México	México	7
8	Ministerio da Saúde	Brasil	7
9	Ministry of Health, La Paz	Bolivia	6
10	Universidad Nacional de Colombia	Colombia	5
11	Universidad de Antioquia	Colombia	5
12	Asociación Colombiana de Infectología	Colombia	5
13	Latin American Society for Travel Medicine (SLAMVI)	Argentina	5
14	Clínica del Country	Colombia	4
15	Clínica Universitaria Colombia	Colombia	4
16	Hospital Albert Einstein	Brasil	4
17	Hospital de Emergencias José Casimiro Ulloa	Perú	4
18	Hospital Infantil de México Federico Gómez	México	4
19	Hospital Universitario San Ignacio	Colombia	4
20	Instituto de Investigaciones Biomédicas, IDB	México	4
21	Instituto de Pesquisa Económica Aplicada	Brasil	4
22	Instituto Nacional de Cancerología	Colombia	4
23	Universidad Autónoma de Nuevo León	México	4
24	Universidad Nacional Autónoma de Honduras	Honduras	4
25	Universidade Federal do Rio de Janeiro	Brasil	4

### Revistas más productivas

En el cuadro 4 se presenta el listado de las revistas más productivas, entre las que se destaca *Travel Medicine Infectious Diseases* (15 documentos). Esta revista se ubica en el cuartil 1 del SJR y está clasificada en las categorías de enfermedades infecciosas y salud pública. La producción científica está concentrada en revistas de los cuartiles 2 y 3, lo que demuestra no solo la alta visibilidad de las contribuciones sino también su posible calidad.

**Cuadro 4:** Distribución de revistas más productivas en el tema de COVD-19 con frecuencia mayor o igual a dos

N°	Revista*	Número de documentos	Cuartil 2018	SJR	Categorías
1	Travel Medicine Infectious Diseases	15	Q1	1,306	Enfermedades infecciosas, salud pública
2	Journal of Medical Virology	7	Q2, Q3	0,966	Infectious diseases, virología
3	Salud Pública de México	6	Q2	0,633	Salud pública
4	Revista de la Sociedad Brasileña de Medicina	5	Q3	0,701	Enfermedades infecciosas, microbiología,
	Tropical				parasitología
5	Cadernos de Saúde Pública	5	Q2	0,585	Medicina, salud pública
6	Revista Epidemiologia e Serviços de Saúde	4	PubMed		Salud pública
7	Le Infezioni in Medicina	4	Q3	0,356	Enfermedades infecciosas, microbiología
8	Jornal Brasileiro de Pneumologia	3	Q3	0,414	Medicina pulmonar y respiratoria
9	Revista Brasileña de Epidemiologia	3	Q3	0,708	Epidemiología
10	Revista de la Facultad de Ciencias Médicas, Universidad Nacional de Córdoba	3	Q4	0,137	Medicina
11	Lancet	2	Q1	15,871	Medicina
12	Revista de Saúde Pública	2	Q1, Q2	0,89	Medicina, salud pública
13	Medwave	2	Q4	0,158	Medicina
14	Jornal de Pediatria	2	Q2	0,693	Pediatría
15	BMJ	2	Q1	1,321	Medicina
16	Research in Social and Administrative Pharmacy	2	Q1	0,876	Ciencia farmacéutica, farmacia
17	Excli Journal	2	Q2, Q3	0,612	Ciencia animal, hallazgo de medicamentos, medicina molecular, farmacología
18	Cureus	2	PubMed		Medicina
19	Annals of the Rheumatic Diseases	2	Q1	7,081	Bioquímica, inmunología, reumatología
20	Infectio	2	Q3	0,164	Enfermedades infecciosas, microbiología, farmacología
21	Archivos Brasileños de Cardiología	2	Q3	0,407	Cardiología

El modelo de dispersión de Bradford describe el comportamiento de la distribución de las revistas en zonas centrales y periféricas según la productividad ^20^. La cantidad de revistas en el núcleo y en las zonas sucesivas permite visualizar las más utilizadas por los investigadores latinoamericanos en el tema objeto de estudio.

**Figura 5:**
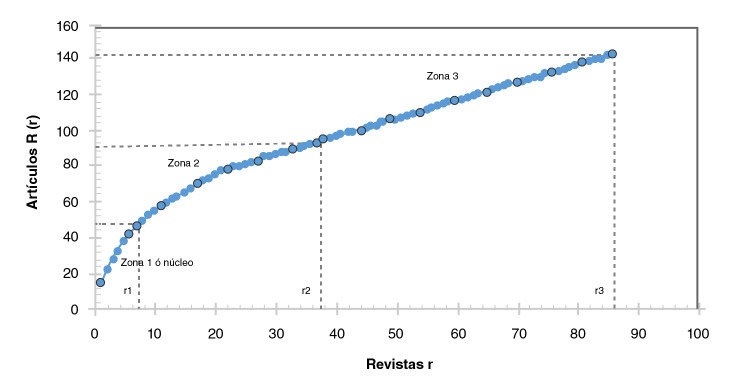
Distribución logarítmica de revistas y documentos por zona según el modelo de Bradford

Siguiendo el proceso metodológico de Bradford, los 142 documentos publicados en 86 revistas. En la zona 1 aparecen siete revistas en las que se ha publicado el 32 % de los estudios. La zona 2 está conformada por 30 revistas (35 %) y la zona 3 por 49 revistas (57 %) para el total de 86 revistas. Como se observa en la figura 5, el crecimiento exponencial de la producción mantiene un ritmo de dispersión hacia la periferia.

### Red de coocurrencia de palabras claves

**Figura 6:**
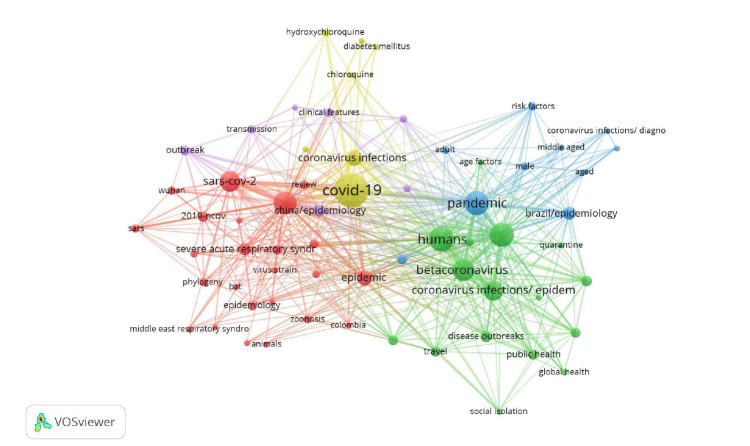
Visualización de una red de aparición concomitante de palabras clave

En la figura 6 se observa que el descriptor de mayor frecuencia es COVID-19 (nombre de la enfermedad), con 79 apariciones, le siguen *humans* y *pandemic,* con 43 y 39 apariciones, respectivamente. En este caso, el número de coocurrencias de dos palabras indica el número de publicaciones en el cual ambas palabras aparecen en la lista de las palabras clave de los documentos seleccionados ^7^.

Nota: se tuvieran en cuenta las palabras con más de dos apariciones. De las 400 palabras clave, solo 64 cumplieron dicho umbral; método de conteo: fractional counting; método de normalización: fuerza de asociación; atracción: 1; repulsión: -3; resolución de agrupamiento: 1,0

Los colores indican agrupaciones de palabras clave relativamente relacionadas entre sí según la fortaleza de asociación obtenida por el programa VOSviewer, además de la diferencia visual de agrupaciones.

Con los 64 descriptores de un total de 400 registrados en los 142 documentos recuperados y los cinco clústers observados, se analizó el enfoque temático de cada agrupación con sus cinco descriptores más importantes según el número de apariciones (cuadro 5). Así, el clúster 1 (rojo) incluye el virus SARS-CoV-2, los primeros nombres como enfermedad del coronavirus 2019 y 2019-ncov, la relación con epidemia y con animales.

**Cuadro 5:** Enfoques temáticos dentro de cada clúster de palabras clave

Clúster	Palabra clave	Apariciones	Enfoque temático
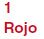	Coronavirus disease 2G19	36	El SARS-CoV-2,
	SARS-CoV-2	28	epidemia y
	Epidemic	16	animales
	Severe acute respiratory syndrome coronavirus 2	10	
	2G19-ncov	8	
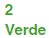	Humans	43	Población, brotes
	Pneumonia, viral	39	de enfermedad
	Betacoronavirus	32	y posibles
	Coronavirus infections/ epidemiology	27	consecuencias del
	Disease outbreaks	9	contagio
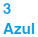	Pandemic	39	Pandemia,
	Brazil/epidemiology	12	incidencia en el
	Virus pneumonia	7	sexo masculino
	Male	5	y los factores de
	Risk factors	5	riesgo
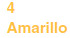	Covid-19	79	El covid-19 y
	Coronavirus infections	18	la relación con
	Hydroxychloroquine	4	tratamientos
	Chloroquine	3	médicos
	Diabetes mellitus	3	
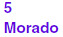	China/epidemiology	10	La enfermedad
	Outbreak	7	vista desde China,
	Clinical features	5	el brote y su
	Transmission	5	reproducción y su transmisión
	Basic reproduction number	4	

El clúster 2 (verde) analiza los temas de población, brotes de la enfermedad, el linaje de los betacoronavirus y las posibles consecuencias del contagio. El clúster 3 (azul) tiene que ver con la pandemia, la incidencia en el sexo masculino, y el estudio de los posibles factores de riesgo. El clúster 4 (amarillo) se enfoca en tratamientos médicos para enfrentar la COVID-19 y, por último, el clúster 5 (morado) abarca los temas relativos al brote, la reproducción y la transmisión de la enfermedad en China.

## Discusión

Aunque incipiente, la producción científica latinoamericana sobre COVID-19 registrada en las bases de datos utilizadas evidencia un ritmo de crecimiento sostenido y exponencial. Este comportamiento es similar al de otras investigaciones recientes, con gran número de documentos publicados ^6^^)^ y ritmos de crecimiento acelerados, incluso en el término de una semana ^3^.

Esta tendencia permite ver la importante participación de las regiones en las investigaciones referentes al tema en un periodo de tiempo reducido, pero, además, un fenómeno importante como la rapidez con la que las revistas científicas se encuentran publicando la investigación sobre COVID-19, con el fin de divulgar en el menor tiempo posible los resultados de los estudios y ofrecer una gran parte de ellos en acceso abierto.

Resultados como la alta colaboración, el liderazgo y la publicación en revistas internacionales reflejan los esfuerzos coordinados en la búsqueda de respuestas a la enfermedad, la gran cantidad de países e instituciones que realizan aportes conjuntos a la investigación mundial mediante el trabajo colaborativo, en una dinámica que revela no solo liderazgos individuales sino el aporte de muchos como la práctica que se impone. La alta tasa de colaboración internacional encontrada en el análisis (52,8 %), resultado de la cooperación en torno al tema, así como la elevada producción con liderazgo, muestra la capacidad de las instituciones regionales de generar investigaciones y aportar recursos a la investigación mundial.

El liderazgo de un importante grupo de autores, instituciones y países en la región con investigaciones relativas a la contribución y la transmisión zoonótica de la COVID-19, su relación con el SARS-CoV-2 y otros aspectos importantes, así como su relación con instituciones de países que lideran la investigación como India, Estados Unidos y Alemania, es más que un resultado de tendencia y visibilidad, pues refleja, además, los aportes de la región a través de instituciones como la Universidad Tecnológica de Pereira, la Fundación Universitaria Autónoma de las Américas y otras de Brasil, México y Colombia. Estos patrones de colaboración en la producción científica latinoamericana sobre COVID-19 siguen los patrones de colaboración internacional usuales en la investigación médica de la región ^21^.

La publicación de los artículos en revistas internacionales (más que en las regionales), en su mayoría de elevada visibilidad e impacto (cuartiles intermedios en el SJR), muestra una tendencia positiva en cuanto a la calidad de las investigaciones. En este sentido, el 8 % de las revistas núcleo cubren más del 30 % de la producción de la región, especialmente la revista *Travel Medicine and Infectious Disease* del Reino Unido, situada en el Q1, con 15 documentos sobre el tema y autores cuya filiación corresponde a instituciones de algunos de los países latinoamericanos. Este resultado es importante si se tiene en cuenta que, como se ha expuesto ^22^, la publicación en revistas con buenos indicadores (citas, impacto, cuartil), supone una mayor cantidad de citas.

Asimismo, la cantidad de países e instituciones dedicadas a la investigación sobre COVID-19 supone que en el mediano plazo aumenten considerablemente los resultados científicos de investigaciones de todo tipo y los artículos producto de las investigaciones. Los resultados que se presentan evidencian que la mayoría de documentos se han publicado en revistas internacionales como resultado de la colaboración internacional, visión que las revistas de nuestros países pueden también adoptar.

El análisis de coocurrencia de palabras o descriptores arrojó cinco agrupaciones y refleja los diferentes enfoques en las investigaciones, lo que clarifica las tendencias de la investigación sobre el tema estudiado. Términos como SARS-CoV-2, el virus causante de la enfermedad, y sus diferentes nombres adoptados, así como los factores de riesgo de la pandemia, los tratamientos necesarios, la transmisión de la enfermedad y las consecuencias del contagio, se relacionan con los resultados de otras investigaciones globales ^23^. Lo importante de este resultado es que la investigación en Latinoamérica se desarrolla en línea con la que se lleva a cabo en los principales países, entre ellos, China, Alemania, Estados Unidos, y otros también mencionados en estudios recientes de corte bibliométrico ^24^.
